# In situ Raman analyses of the soot oxidation reaction over nanostructured ceria-based catalysts

**DOI:** 10.1038/s41598-019-39105-5

**Published:** 2019-03-07

**Authors:** Enrico Sartoretti, Chiara Novara, Fabrizio Giorgis, Marco Piumetti, Samir Bensaid, Nunzio Russo, Debora Fino

**Affiliations:** Department of Applied Science and Technology, Politecnico di Torino, Corso Duca degli Abruzzi, 24, 10129 Turin, Italy

## Abstract

To reduce the emissions of internal combustion engines, ceria-based catalysts have been widely investigated as possible alternatives to the more expensive noble metals. In the present work, a set of four different ceria-based materials was prepared via hydrothermal synthesis, studying the effect of Cu and Mn as dopants both in binary and ternary oxides. *In situ* Raman analyses were carried out to monitor the behaviour of defect sites throughout thermal cycles and during the soot oxidation reaction. Despite ceria doped with 5% of Cu featured the highest specific surface area, reducibility and amount of intrinsic and extrinsic defects, a poor soot oxidation activity was observed through the standard activity tests. This result was confirmed by the calculation of soot conversion curves obtained through a newly proposed procedure, starting from the Raman spectra collected during the *in situ* tests. Moreover, Raman analyses highlighted that new defectiveness was produced on the Cu-doped catalyst at high temperature, especially after soot conversion, while a slight increase of the defect band and a total reversibility were observed in case of the ternary oxide and pure/Mn-doped ceria, respectively. The major increment was related to the extrinsic defects component; tests carried out in different atmospheres suggested the assignment of this feature to vacancy-free sites containing oxidized doping cations. Its increase at the end of the tests can be an evidence of peroxides and superoxides deactivation on catalysts presenting excessive oxygen vacancy concentrations. Instead, ceria doped with 5% of Mn exhibited the best soot oxidation activity, thanks to an intermediate density of oxygen vacancies and to its well-defined morphology.

## Introduction

Nowadays gasoline and diesel engines are still the most used propulsion systems for both trucks and passenger cars. Internal combustion engines emissions are among the causes of the rise in air pollution. Among the air pollutants emitted by these sources, CO and particulate matter (PM) are critical, as they can cause several health problems, such as respiratory and cardiovascular diseases or even cancer^1–3^.

To counteract the increase in pollution, many countries have introduced emission limits for all vehicles, and over the years the regulations have become increasingly strict, and have imposed the adoption of post-treatment systems for gas exhaust on all vehicles^4^. Nowadays Diesel vehicles, which are appreciated for their great efficiency and fuel economy, are equipped with a particulate filter that can entrap fine dusts. This filter must be periodically regenerated, and 600 °C should be reached to cause the combustion of carbon particles, while the exhaust gas temperature of a Diesel engine is usually around 200–500 °C^5,6^. The use of catalysts dispersed on the filter walls allows to lower the regeneration temperature, reducing fuel consumption and the associated CO_2_ emission, besides avoiding excessive thermal shock for the materials^7,8^.

To this end, mixed oxide catalysts are widely studied as lower costs alternatives to noble metals. Many studies have been focused on ceria-based materials, used in three-way catalysts or as catalyst supports, since they offer a good activity due to their ability to release or absorb oxygen atoms, which confers them a high oxygen storage capacity (OSC)^9–13^. The ability of a catalyst to lower the temperature required for particulate combustion does not only depend on its intrinsic activity; actually, since the reaction involves two solid phases, the efficiency of contact between the catalyst and the particulate also plays a fundamental role^14,15^. For this reason, many advances have been made in the synthesis of nanostructured ceria-based catalysts, whose reactivity depends not only on the particle size but also on the crystalline planes exposed on the surface, as pointed out in several studies^11,16,17^. In particular, the oxygen reactivity is thought to be higher on the less stable facets, and it is supposed to follow this trend among the low-index surfaces: (111) < (110) < (100)^18^. Another factor that generally affects the activity of a catalyst and its OSC is the presence of surface defects, e.g. oxygen vacancies^19,20^. The vacancy formation energy depends on the type of exposed facets, and it is believed to conform to this trend: (110) < (100) < (111)^18^. To increase the number of surface defects and increase ceria activity, dopant atoms can be inserted into the crystalline lattice, and this often also provides the material with greater thermal stability, increased oxygen mobility and reducibility of cerium atoms^21^. Both rare earth elements and transition metals can be used as dopants^22,23^; for instance, previous studies have demonstrated the positive effect of Cu or Mn addition, ascribed to the coupled redox cycles between cerium and copper or manganese ions^24–27^.

Thanks to its remarkable sensitivity to the microstructural evolution of the analyzed samples, Raman spectroscopy has become widespread in recent years in various research fields concerning materials science, including catalysis. Regarding ceria-based catalysts, this technique has been used to investigate the interaction of molecules with their surface or the defects present in the lattice^28^. Indeed, defect sites have been found to influence ceria catalytic activity, playing an important role in catalysis^20^. However, up to now the evolution of defects with temperature in doped systems and during the catalytic activity has been poorly investigated. In particular, monitoring the defect sites throughout the soot oxidation reaction presents several complexities^29^ and only few observations of this process have been reported^30^.

In this work, four nanostructured ceria-based catalysts (CeO_2_ nanocubes, 5% Cu doped CeO_2_, 5% Mn doped CeO_2_, 2.5% Cu and 2.5% Mn doped CeO_2_) prepared through hydrothermal synthesis^31^ were studied by *in situ* Raman spectroscopy at high temperature and during soot oxidation. Temperature-dependent Raman analyses were performed on the catalysts, studying the evolution of the identified defect sites in different atmospheres. The performance of the ceria-based NPs in soot oxidation was then investigated and the results compared to standard activity tests, through the calculation of Raman based soot conversion curves. The role of the different defect sites was discussed, analyzing their behavior in terms of the proposed mechanisms for soot oxidation.

## Results and Discussion

### Catalyst characterization

**Figure 1:**
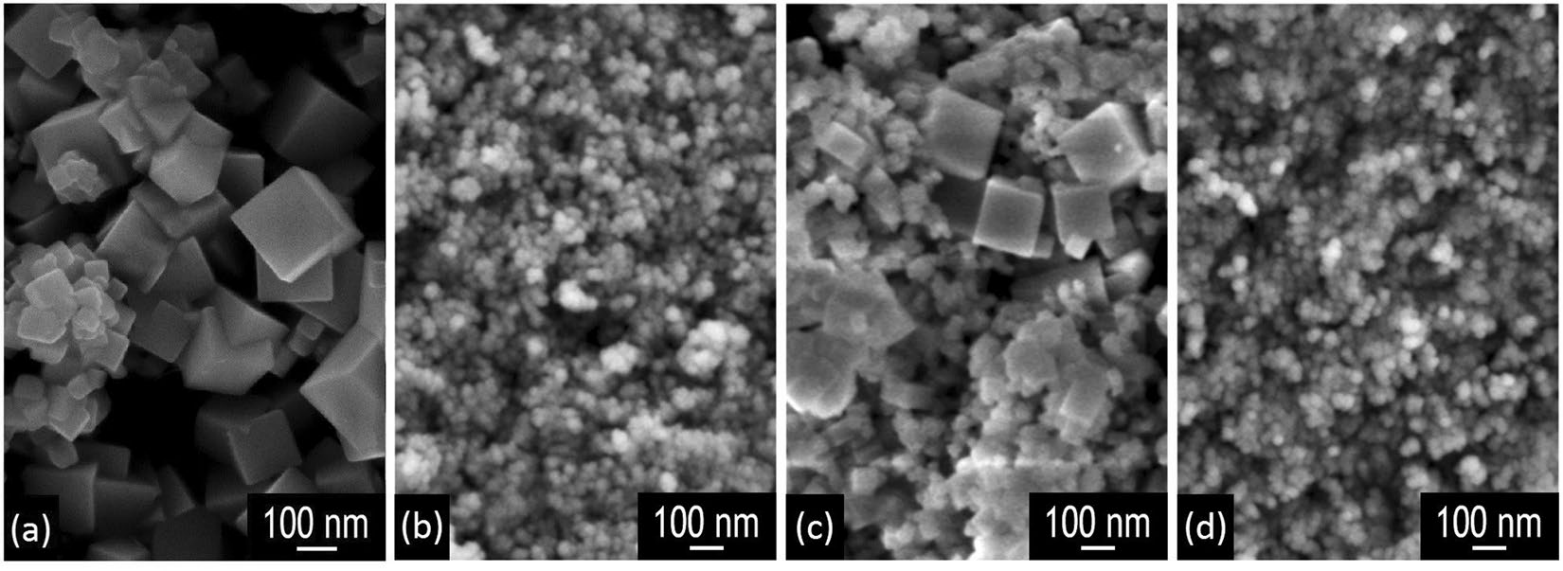
FESEM micrographs of the CeO_2_ (**a**), Ce95Cu5 (**b**), Ce95Mn5 (**c**) and Ce95Cu2.5Mn2.5 (**d**) catalysts.

The synthesized catalysts were first characterized by complementary techniques in order to investigate their morphology, crystalline structure, surface area and surface species; some of the results obtained were previously reported elsewhere^31^ (gathered in Table 1), but are briefly resumed here to present a complete description of the investigated materials. Figure 1 shows the FESEM (Field Emission Scanning Electron Microscopy) micrographs of the four catalysts. The CeO_2_ sample presents a well-defined nanocubic morphology, with the size of the cubes ranging from 100 to 300 nm and many (1 0 0) planes exposed^32,33^. The presence of nanocubes has been linked to better performances in the catalytic oxidation of both carbon monoxide and particulate matter^11,12,34^; concerning the latter reaction, the effect of morphology is more evident, since the shape of ceria nanoparticles affects directly the density of contact points between the catalyst and soot^35^. Also the 5% Mn doped catalyst (Ce95Mn5) maintains a morphology similar to that of CeO_2_, but with smaller nanocubes, whose size ranges from 50 to 100 nm. On the contrary, when copper is introduced into ceria lattice, the nanocubes disappear. Both the 5% Cu doped (Ce95Cu5) and 2.5% Cu, 2.5% Mn doped (Ce95Cu2.5Mn2.5) catalysts are formed by small particle agglomerates, probably consisting of nanoparticles characterized by high-index planes, like nanopolyhedra^36^; for both of the samples, the nanoparticles have average sizes between 20 and 40 nm.

**Table 1:** Some properties of the synthesized catalysts obtained by XRD, N_2_-physisorption at −196 °C and XPS (complete catalysts characterization, except for Ce95Mn5, reported in^31^).

Catalyst	*D*_c_ (nm)	*S*_BET_ (m^2^/g)	*V*_p_ (cm^3^/g)	*D*_p_ (nm)	[O_α_] (% at.)	[O_β_] (% at.)	[Ce^3+^] (% at.)
CeO_2_	135	9	0.03	12	31	69	21
Ce95Cu5	34	46	0.16	14	29	71	27
Ce95Mn5	79	17	0.05	12	33	67	21
Ce95Cu2.5Mn2.5	23	52	0.15	11	40	60	26

Crystallite size (*D*_c_) was also investigated through XRD (X-Ray Diffraction) analyses, and the *D*_c_ values calculated with the Scherrer’s equation, reported in Table 1^31^, are in good agreement with the FESEM observations. Mixed oxide samples have a smaller *D*_c_ than pure CeO_2_, and the two Cu-doped catalysts present the lowest *D*_c_ values: this suggests a major change in the crystalline structure due to the dopant incorporation, which is in agreement with the strong interaction between ceria and copper that has been widely discussed in the literature^25,37^. However, all the powder XRD diffractograms (reported in Supplementary Fig. S1) exhibit the typical pattern of the crystalline fluorite structure of ceria.

Previous results are in accordance with those obtained from N_2_-physisorption, reported in Table 1^31^. The pure ceria sample has the lowest specific surface area, while this is definitely higher for all the doped catalysts. In particular, copper seems to have a greater tendency to increase S_BET_, and this can be explained considering the lower crystallite size of the Ce95Cu5 and Ce95Cu2.5Mn2.5 samples revealed by the XRD and FESEM results. The total pore volume V_p_ follows the same order of the specific surface area, while the average pore diameter D_p_ presents comparable values for the four samples.

Surface oxygen species of the four catalysts were investigated by analysing the O 1 *s* XPS (X-ray Photoelectron Spectroscopy) spectra (shown in Supplementary Fig. S2a). Their deconvolution allows to estimate the amount of capping oxygen species on the surface, the so-called “O_α_”, compared to the quantity of “O_β_” oxygen atoms in the ceria lattice^38,39^; the relative abundances of these two oxygen species in the four catalysts are reported in Table 1^31^. The CeO_2_, Ce95Cu5 and Ce95Mn5 samples present similar relative quantities of O_α_ species. Instead, in the Ce95Cu2.5Mn2.5 catalyst, the quantity of O_α_ is significantly higher. An increase of O_α_ atoms can favor oxygen spillover at solid surfaces.

The presence of reduced Ce^3+^, often associated with oxygen vacancies, was instead investigated by the analysis of Ce 3*d* XPS spectra. Eight peaks can be identified; only the *v*_1_ and *u*_1_ peaks are ascribed to Ce^3+^ species, while the other peaks are associated with the presence of Ce^4+^ species^23,40^. Through the deconvolution of the peaks, the relative abundance of the Ce^3+^ species in the four synthesized catalysts can be estimated; such values are summarized in Table 1^31^. The CeO_2_ and Ce95Mn5 catalysts have similar relative amounts of Ce^3+^ species on the surface, while the Ce95Cu5 and Ce95Cu2.5Mn2.5 samples contain higher percentages of Ce^3+^. This observation suggests that copper addition promotes the formation of Ce^3+^ and redox sites on the surface more than manganese insertion; moreover, this effect is already evident for low Cu percentages, since there are minimal differences (1%) between Ce95Cu5 and Ce95Cu2.5Mn2.5 samples.

The XPS Cu 2*p* and Mn 2*p* spectra are not informative, since the low quantities of doping elements (5% or 2.5%) are associated with peaks characterized by very low intensities and hard to be observed or deconvoluted.

### Raman analyses

#### Analyses at room temperature

Raman spectroscopy can provide useful information about the influence of dopants on ceria structure and on the formation of new defective sites. Raman spectra recorded on ceria-based materials can show several peaks related to both bulk ceria vibrational modes and surface defects. Some of the most significant and sometimes controversial assignments provided by the literature are reported in Table 2.

**Table 2:** Some peak assignments of Raman spectra recorded on ceria-based catalysts at RT.

Raman shift [cm^−1^]	Assignments of the peaks
404	Shoulder due to distortion in the lattice^28^
460–465	Symmetric stretching of the Ce-O_8_ crystal unit (F_2g_ mode), characteristic of the fluorite lattice structure^28,31,41–54^
487	Shoulder due to distortion in the lattice^28^
540	Defect spaces which include an O^2−^ vacancy, observed when 3^+^ dopant cations are introduced in the CeO_2_ lattice^47^
	Extrinsic oxygen vacancy complexes^53^
550	Oxygen vacancies^31,48,54^
	Extrinsic oxygen vacancies^44,51^
560	Oxygen vacancies^41,45,50^
570	Oxygen vacancies in pure and cation-doped ceria^42,49^
580	Intrinsic oxygen vacancies^43^
590–600	Defect spaces including a dopant cation in 8-fold coordination of O^2−^, without any O^2−^ vacancy^41,44,47,48^
	Vacancy-interstitial Frenkel-type oxygen intrinsic defects in ceria^28,31,49,50^
	Oxygen vacancies and reduced Ce^3+^ cations in the ceria lattice^46,51–54^
620	Extrinsic MO_8_ sites capable of delivering oxygen under reducing conditions i.e. part of a Frenkel defect^46^
630	Extrinsic defects band^31^

**Figure 2:**
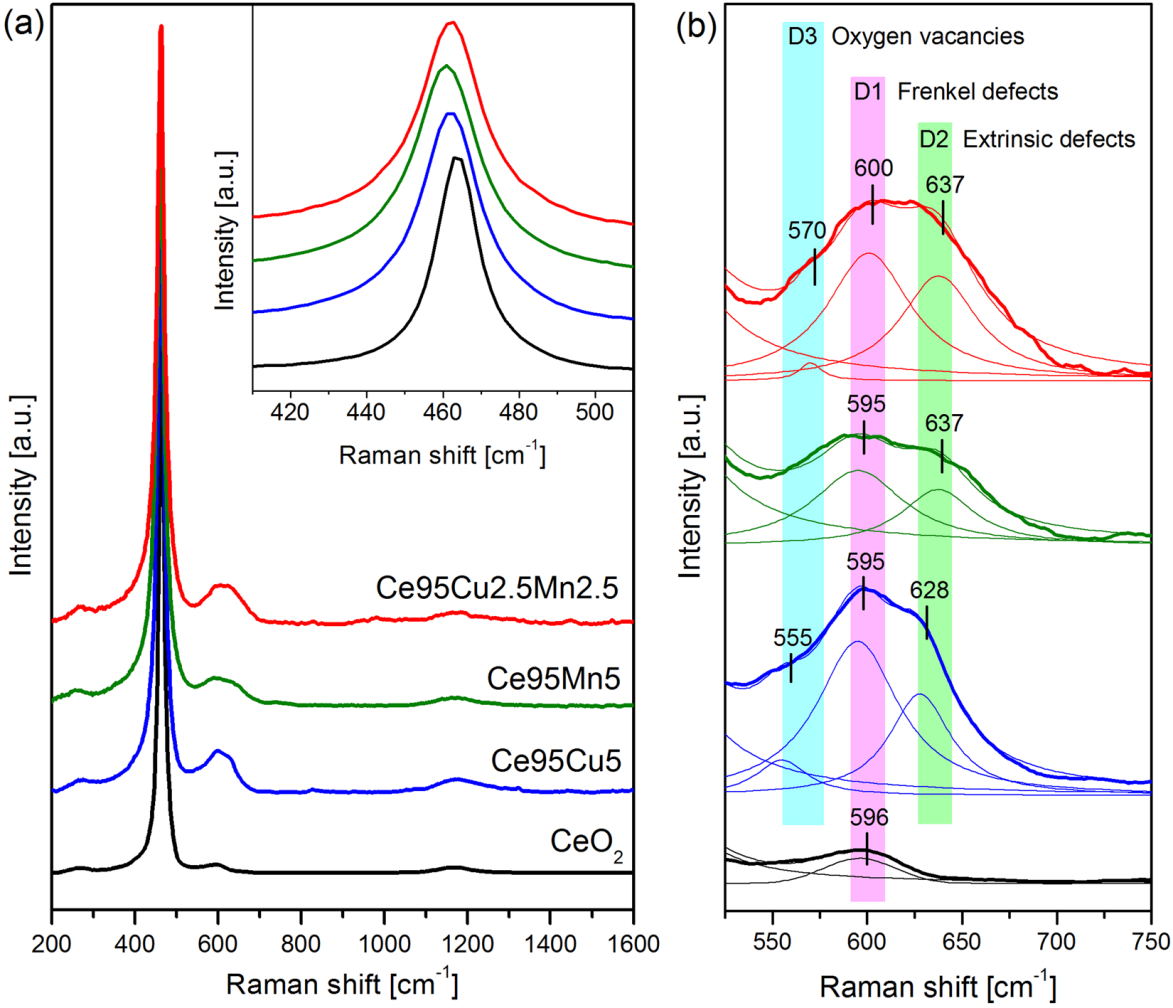
Average Raman spectra of the four catalysts at RT (**a**), with magnifications of the F_2g_ peak (inset) and of the defects band region (**b**). The traces obtained by curve fit and deconvolution of the defect-induced band are also reported (thin lines). All the spectra were normalized to the F_2g_ band.

Figure 2a shows the Raman spectra of the four different samples collected at room temperature (RT), each obtained from the average of three spectra recorded in different points of the sample. All the spectra have been normalized to the most intense peak, namely the F_2g_ peak, located at about 464 cm^−1^ and ascribed to the symmetric stretch mode of the Ce-O_8_ crystal unit, characteristic of the fluorite lattice structure typical of ceria-based materials^28,31,41–54^. The absence of the characteristic signatures of Mn and Cu oxides in Raman spectra indicates that the doping elements are well integrated into the ceria lattice, confirming the XRD results. However, the addition of dopants causes several changes in ceria microstructure, which can be noticed in Raman spectra.

Besides a red-shift and a broadening of the F_2g_ band (inset of Fig. 2a), which may depend on the increase of topological disorder and on a higher defectiveness^55^_,_ new defect-induced bands can be identified in the 500 to 700 cm^−1^ range; Fig. 2b shows a magnification of this region. CeO_2_ spectrum presents a single peak located at 596 cm^−1^, previously assigned by different authors to oxygen vacancies dealing with a Frenkel anion pair, in which an oxygen atom has moved into an octahedral interstitial position generating a vacancy^28,31,49,50^. Other authors ascribed such band to oxygen vacancies associated to the presence of reduced Ce^3+^ cations^46,51–54^, or to defect spaces including a dopant cation without any O^2−^ vacancy^41,44,47,48^. This vibrational feature, which from now on will be referred to as “D1”, can be considered anyway “intrinsic”, i.e. already present in the structure of pure ceria.

For the doped catalysts, the disorder-associated band becomes broader and more intense, and it can be deconvoluted into two different peaks. Besides the already mentioned D1, a “D2” component can be identified, centered at 628 cm^−1^ for the Ce95Cu5 sample and at 637 cm^−1^ for the Ce95Mn5 and Ce95Cu2.5Mn2.5 samples. Since the position of this band depends on the doping element, it is probably linked to “extrinsic” defects, generated by the dopants addition. In some previous literature reports this Raman component has been ascribed to the presence of MO_8_ units without associated oxygen vacancies, where M is a foreign cation^41,46^. Interestingly, in presence of dopants, also the D1 peak at 595 cm^−1^ becomes more intense, suggesting that the addition of the doping elements causes an increase in the amount of both intrinsic and extrinsic defects. Finally, only in the Cu-containing samples a third “D3” peak can be detected, centered at about 555–570 cm^−1^ and much weaker than the other two. This component is usually assigned to oxygen vacancies coupled with the presence of Ce^3+^ or other aliovalent cations^44,48,51,54^. The presence of this peak only in Ce95Cu5 and Ce95Cu2.5Mn2.5 spectra can be linked to the higher abundance of Ce^3+^ in these samples, proved by XPS results reported in Table 1^31^. It should be noted that a closer inspection of the defect-induced band reveals that each identified component is the result of the convolution of more bands, which cannot be however clearly resolved and account for the heterogeneous environment in which a particular defect type can be found.

The defects amount in the four samples was estimated calculating the ratios between the area of the D1, D2 or D3 peak and the one of the F_2g_ band; these parameters, called D1/F_2g_, D2/F_2g_ and D3/F_2g_, respectively, are summarized in Table 3, together with their sum, D/F_2g_. The data reported in Table 3 confirm that the addition of dopants fosters both D1 and D2 defects formation. The two Cu-doped samples show the highest D/F_2g_ ratios, due to a greater defectiveness accompanied by their higher specific surface area. The major abundance of defects in these materials is probably an effect of the strong interaction between ceria and copper, which fosters microstructural changes, and it is affected also by the type of planes exposed by the nanoparticles^56^.

**Table 3:** Raman parameters calculated at RT for the four catalysts.

Catalyst	D1/F_2g_	D2/F_2g_	D3/F_2g_	D/F_2g_
CeO_2_	0.022	—	—	0.022
Ce95Cu5	0.156	0.077	0.023	0.256
Ce95Mn5	0.078	0.046	—	0.124
Ce95Cu2.5Mn2.5	0.116	0.096	0.013	0.231

Several papers report that the D/F_2g_ ratio order reflects well the trend of the catalytic activity for the CO and other gas phase oxidation reactions^22,37,57^. Such correlation was previously observed also for the analyzed set of catalysts^31^, suggesting that the presence of a greater number of defects promotes the conversion of CO to CO_2_; indeed the specific reaction rates of CO conversion increased according to the order CeO_2_ < Ce95Mn5 < Ce95Cu2.5Mn2.5 < Ce95Cu5, in analogy to the D/F_2g_ ratio and the specific surface areas. Instead, the trend of catalytic activity for the soot oxidation reaction is usually different, suggesting that other factors, such as the catalyst morphology or the contact points between catalyst and soot, play a more important role; in particular, ceria nanocubes and nanorods usually exhibit better performances when compared to nanopolyhedra, spindles or mesoporous polycrystalline ceria samples^11,36,58^.

#### Static analyses at high temperature

**Figure 3:**
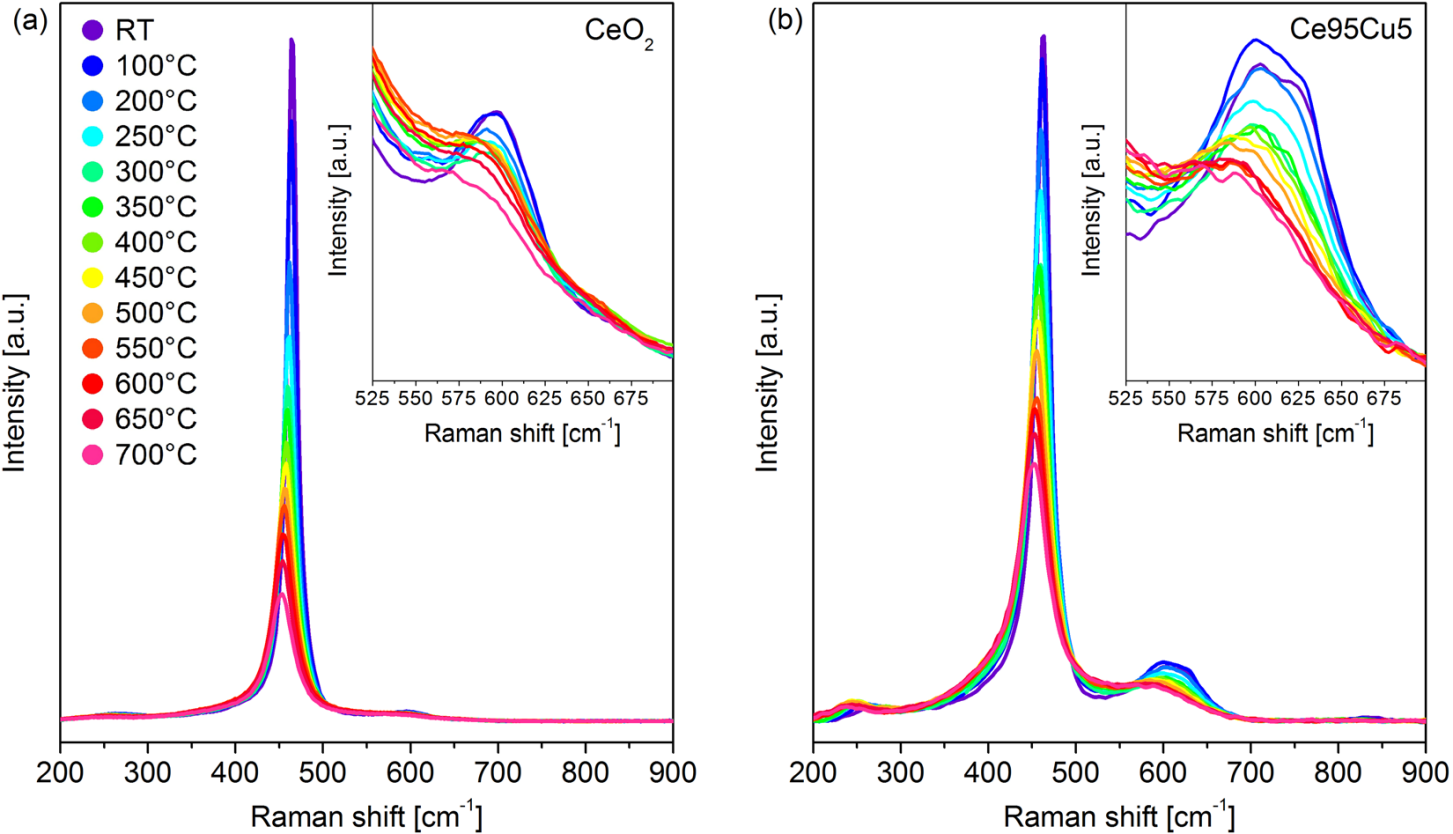
Raman spectra collected at different temperatures on the CeO_2_ (**a**) and Ce95Cu5 (**b**) samples during the static tests. The inset shows a magnification of the defects region.

The four catalysts were analyzed in the 25–700 °C range in order to monitor the evolution of their vibrational spectral features at the catalytic temperatures. Figure 3a shows the Raman spectra of the CeO_2_ sample acquired in air under a static atmosphere. As the temperature increases, the Raman peaks move to lower Raman shift and their width increases, while their intensity decreases. Both the position and the width of the peaks vary almost linearly with the temperature. The red-shift of the peaks is mainly due to thermal-induced strain; the spectral broadening and the intensity quenching are instead due to anharmonicity effects^46,59^. The temperature, however, affects differently the intensity of the Raman bands for collective and more localized modes: for this reason, it is not possible to determine if the amount of defects increases or decreases by comparing the ratios between the areas of the peaks calculated at different temperatures, as the comparison could be unreliable^60^. Similar effects are observed on the doped catalysts, as shown in Fig. 3b for Ce95Cu5. An unresolved D band is detected at high temperatures, due to the broadening of the three components.

**Figure 4:**
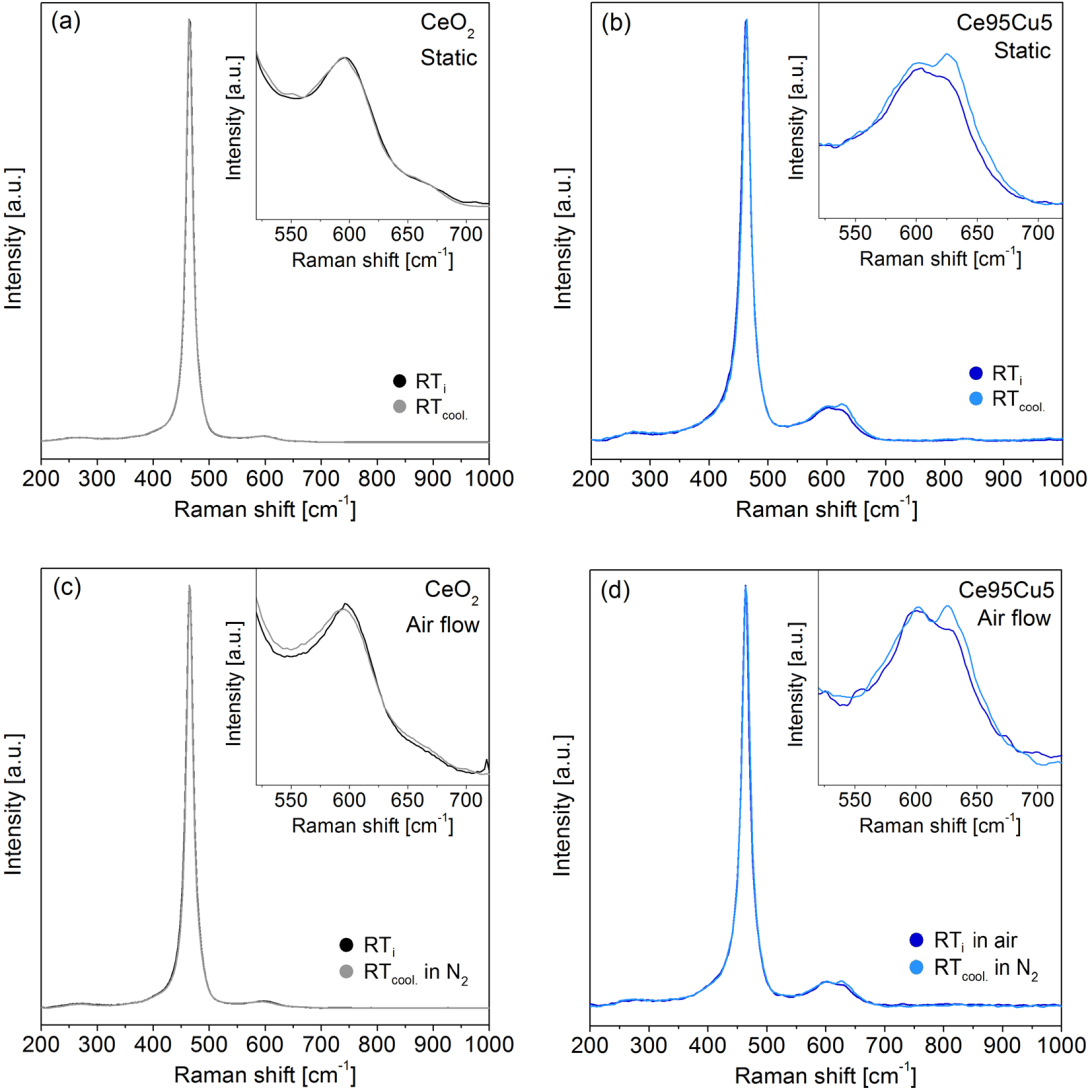
Comparisons between the Raman spectra collected on CeO_2_ (**a**) and Ce95Cu5 (**b**) at RT at the beginning (RT_i_) and at the end (RT_cool._) of the static tests, in which the samples were heated up to 700 °C and then cooled down to RT in a static air atmosphere; the same comparisons are reported also for the analyses in air flow (with cooling ramp in N_2_ flow) on CeO_2_ (**c**) and Ce95Cu5 (**d**). All the spectra were normalized to the F_2g_ band. The insets display a magnification of the defects region.

After reaching 700 °C, the samples were cooled to RT. During the cooling phase, two spectra were collected at 350 °C (350 °C_cool._) and 25 °C (RT_cool._). For what concerns CeO_2,_ the spectra recorded at the same temperatures during the heating and cooling ramp are almost overlapped, as shown in Fig. 4a, where the comparison between the RT_cool._ and RT_i_ is reported (see Supplementary Fig. S3a for the 350 °C_cool._ spectra). This correspondence suggests that any structural change caused by the heating is completely reversible: thus, there is no defects evolution after thermal cycles. This could be a further confirmation of the assignment of the D1 peak to Frenkel defects, which are generally rather stable with the temperature^50^. A similar reversible behavior was observed for the two Mn-doped catalysts. On the other side, for the Ce95Cu5 sample the two spectra recorded at 350 °C overlap (Supplementary Fig. S3b), but the ones registered at RT at the beginning and at the end of the test are quite different (Fig. 4b). While the F_2g_ band is unaffected by the thermal cycle, the defect band reveals a spectral change: in detail, the D1 component remains almost unchanged, but the D2 peak moves slightly to higher Raman shift and becomes more intense. This result points out an unexpected behavior for the Ce95Cu5 catalyst, as previous literature works report either a decrease of the D/F_2g_ ratio due to particle aggregation or a reversibility of the Raman spectra after thermal cycles^45,46,60^. The variation can be quantified by comparing the D2/F_2g_ ratio of the initial and final spectra: this parameter increases of 34% during the test. It can be therefore inferred that there is actually an increase in the number of D2 defects in ceria lattice during heating and that these changes are not completely reversible once returned to RT. The oxygen-rich atmosphere in this test suggests however that the formed species are related to the presence of Cu in high oxidation states, namely Cu^2+^ or even Cu^3+^, which was detected by XANES and proposed as part of the catalytically active site in CO oxidation by Elias *et al*.^61^.

#### Analyses in air flow at high temperature

Once the temperature-dependent spectra were acquired in a static atmosphere by allowing the system to reach the thermal equilibrium at each step, the actual conditions used in the catalytic processes were tested. The thus obtained Raman spectra are very similar to those recorded during the static analyses. Although in this case the sample was kept in air flow and heated following a temperature ramp, no significant differences could be detected. An additional test was set-up in order to understand if any change involving the defect sites at high temperature could be preserved at the end of the cooling ramp. After recording the spectrum at 700 °C, pure nitrogen was fluxed into the cell. After fifteen minutes a new spectrum was acquired at 700 °C, and no differences due to the change of the atmosphere were detected.

The whole cooling phase was then conducted in nitrogen flow. Once returned to RT, a spectrum was acquired, which can be compared with the RT_i_ one. In the case of the CeO_2_ (Fig. 4c), only a slight increase of the D band width was observed. Such change could be related to the appearance of an extremely weak shoulder due to the formation of Ce^3+^-associated oxygen vacancies (D3 band), but the very small difference does not allow conclusive explanations. The same broadening was not observed for the Ce95Mn5 and Ce95Cu2.5Mn2.5 samples, whose RT_i_ and RT_cool._ spectra are almost perfectly overlapped, as happened for the static tests. This suggests that very few new oxygen vacancies are generated at high temperature in the absence of a reductant to be converted; indeed, the absence of oxygen in the atmosphere during the cooling-down should hamper its re-adsorption from the gaseous phase and the refilling of oxygen vacancies generated at high temperature, leading to an irreversible increase of the vacancy related band (D3), which is actually not clearly observed. Also for the Ce95Cu5 sample, the comparison between the initial and final spectra recorded at RT, shown in Fig. 4d, leads to results similar to those previously obtained with the static analysis. Once again, a change in the defect region can be detected, since the intensity of the D2 component increases: the D2/F_2g_ ratio varies from 0.061 at the beginning of the test to 0.083 at the end, with a 35% increase, similar to that observed in the static analysis. The absence of any effect of the nitrogen atmosphere means that the new defect sites are generated during the heating ramp and confirms their assignment to oxidized copper sites. Their formation in the early stages of the heating process is also supported by the perfect overlapping of the 350 °C spectra, recorded during the heating and cooling phases. As the presence of Cu^+^ cations is often associated with an oxygen vacancy in the lattice, it could be hypothesized that oxygen vacancy filling occurs during Cu sites oxidation, and therefore that the D2 component is actually due to a vacancy-free defect site. The suggested assignment is further supported by an additional heating and cooling ramp in nitrogen atmosphere, at the end of which the increase of the D2 component and the overall raise of the D/F_2g_ ratio are negligible (Supplementary Fig. S4). Moreover, RT exposure of the catalyst to air after the treatment readily led to an increase of the D2 component, pointing out the need of an oxidizing atmosphere to allow the formation of the discussed defect species.

#### *In situ* analyses of soot conversion

**Figure 5:**
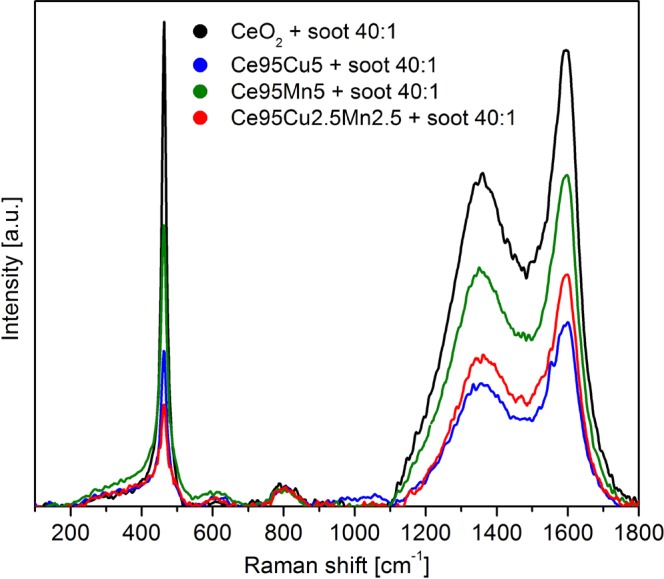
Raman spectra collected at RT on the four tablets of mixed catalyst and soot.

Figure 5 shows the Raman spectra recorded at RT on the four tablets prepared by mixing catalyst and soot in tight contact. When soot is added to the catalyst, in the Raman spectra two large partially overlapping bands appear, centered at about 1350 cm^−1^ and 1600 cm^−1^, typical of amorphous carbon^62,63^. Besides these, other two minor bands are detected: one is centered at about 800 cm^−1^, while the second extends in the range 200–450 cm^−1^, partially superimposed to the F_2g_ peak; such features are typical of silica, which is present in Printex U as a contamination (see Supplementary Fig. S5). Despite both the F_2g_ peak and the defects band of the catalyst are still visible, the Raman signal is weak due to the huge absorption of light by soot particles. The resulting high noise does not allow a reliable interpretation of the shape of the defect bands at RT.

**Figure 6:**
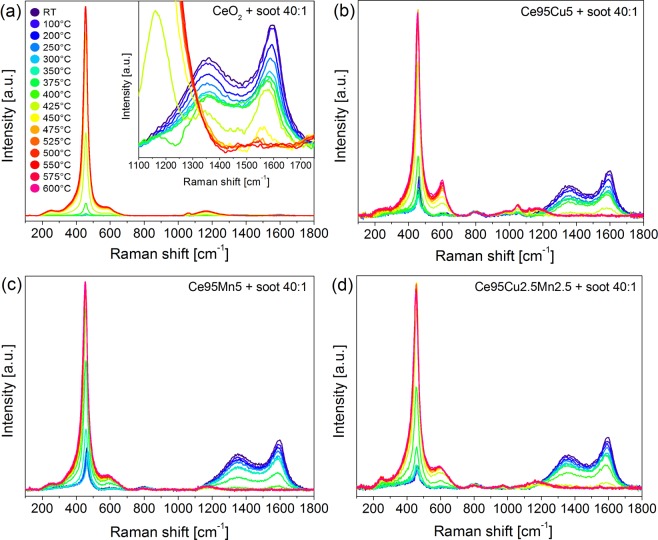
*In situ* Raman spectra recorded at different temperatures during the analysis of soot oxidation on the four tablets: CeO_2_ (**a**), Ce95Cu5 (**b**), Ce95Mn5 (**c**) and Ce95Cu2.5Mn2.5 (**d**). The evolution of the soot Raman bands is magnified in the inset of Fig. 6a.

Figure 6 shows the Raman spectra recorded during the *in situ* tests on the four tablets of catalyst and soot. Generally, at the beginning of the test, all the Raman peaks, including the typical vibrational features of soot, move to lower Raman shift and become larger and less intense as the temperature increases, due to the effect of heating on the vibrational properties of the materials, as detailed above. At a certain temperature, soot conversion starts and soot-related peaks quickly lose intensity; meanwhile all the typical modes of ceria become more intense due to the reduction in light absorption caused by the decrease in the soot content in the tablets. Once soot is totally converted, the peaks of the catalysts begin to lose intensity again.

**Figure 7:**
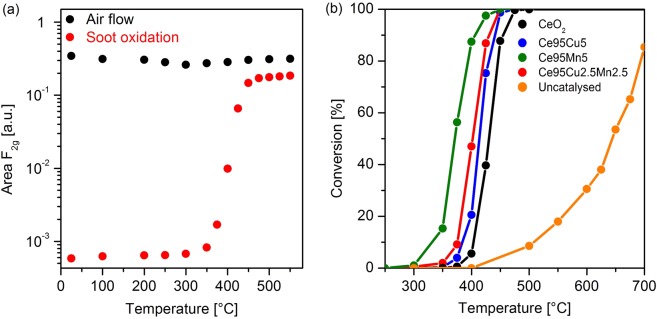
Variation of the F_2g_ peak area as a function of temperature during the analysis in air flow (black dots) and during soot oxidation (red dots) on the CeO_2_ catalyst (**a**); the area of the F_2g_ band has been normalized to the acquisition parameters and to the area of the main band of the internal Si reference. Soot conversion curves obtained from the Raman spectra collected during the *in situ* tests over the four catalysts and for the uncatalyzed reaction (**b**); the last curve was obtained as detailed in the caption of Supplementary Fig. S6.

Supplementary Fig. S6 reports the result of the same measurements performed on a 40:1 silica-soot tablet in air as a control sample. The complete conversion of soot in air in the absence of the catalyst is attained at temperatures around 750 °C, confirming the improved soot oxidation in presence of the catalytic materials. In detail, Ce95Mn5 appears to be the most active catalyst in the series, in agreement with the standard catalytic activity tests results reported in Supplementary Fig. S7. Soot conversion starts at 350 °C and is complete at 425 °C, as observed by the disappearance of the carbon-related Raman bands. Pure ceria and Cu-doped samples are characterized by worse performances. The results can be better analyzed by calculating a conversion curve starting from the Raman spectra. In standard activity tests, the conversion is obtained as the ratio between the amount of CO_2_ produced at a certain temperature and the total amount measured during the whole oxidation process by IR absorption. In analogy, the area of the main Raman soot band could be employed for the calculation, but the decrease of its intensity due to the heating complicates the identification of the onset of the catalytic activity. Instead, as shown in Fig. 7a, the variation of the F_2g_ band intensity vs. temperature clearly differs in presence of soot and the light off of the reaction can be therefore detected by the deviation from its normal trend. The intensity change covers more than one order of magnitude for all the catalysts, allowing a very good sensitivity, which is also confirmed by the fact that a slight increase of the band intensity is still observed when all the soot Raman features have disappeared. The conversion curves, shown in Fig. 7b, are therefore calculated through the equation (1), as the ratio between the variation of the integrated area of the F_2g_ band at a certain temperature (T_x_) with respect to the catalytic process onset one (T_i_) and the total area increase measured at the temperature at which the maximum intensity is reached, before the F_2g_ band starts to decrease again (T_f_):

1 $$Soot\,conversion=\frac{{A}_{F2g}({T}_{x})-\,{A}_{F2g}({T}_{i})}{{A}_{F2g}({T}_{f})-\,{A}_{F2g}({T}_{i})}$$

It should be noted that the Raman curves are shifted toward lower temperatures in comparison to the ones calculated in the catalytic activity tests (reported in Supplementary Fig. S7a). This phenomenon can be partially explained by the lower soot-catalyst ratio employed in the Raman measurements, in order to preserve an acceptable signal-to-noise ratio. Indeed, Supplementary Fig. S8 reports the soot conversion for a 9:1 CeO_2_-soot tablet; during this test, an increase of almost 20 °C of the T_50%_ (temperature at which the reaction reaches 50% of the soot conversion) was observed, due to the higher soot content. The Raman conversion curves confirm the high activity of the Ce95Mn5 catalyst, and also the similar lower activities of Ce95Cu5 and Ce95Cu2.5Mn2.5. However, the behaviour of CeO_2_ is quite different. Indeed, its conversion curve is delayed with respect to the ones of the doped ceria samples. In agreement with this result, soot Raman bands are still observed at higher temperatures compared to the other catalysts. The repetition of both the standard and Raman tests pointed out the reproducibility of these outcomes, thus showing that slight variation of the soot concentration in the analyzed laser spot cannot account for this discrepancy.

Actually, in previous studies the effect of the catalyst optical absorption on the penetration depth at specific laser wavelengths and therefore on the observed Raman features has been carefully analyzed^64^. It can be reasonably postulated that such effects can explain the unexpected trend in the Raman conversion. Indeed, the doped catalysts provide similar higher light absorption (i.e. lower penetration depth) at the 514.5 nm excitation wavelength with respect to the pure ceria (see Supplementary Fig. S9). As in the set-up of the heating cell air is flowing on the top of the catalyst tablet, a slightly delayed soot oxidation is expected for the deepest layers, which can be only probed in the case of the least absorbing CeO_2_ catalyst. The dependence of the results on the absorption features of the sample, which can differ significantly from one material to another, constitutes a limitation of the proposed method, since a quantitative correction taking into account the material absorption coefficient is not easily available. The comparison of Fig. 7b and Supplementary Fig. S7a also reveals a swap between the Ce95Cu5 and Ce95Cu2.5Mn2.5 conversion curves calculated from the Raman data, which can also be ascribed to the shorter penetration depth of the exciting laser light into the Ce95Cu2.5Mn2.5 tablet. Indeed, the UV-Vis diffuse reflectance spectra in Supplementary Fig. S9 suggest a higher light absorption for the ternary oxide catalyst, confirming that such measurements can provide a good qualitative explanation of the discrepancies observed between the conversion curves obtained by the two procedures. The Raman conversions could better match the standard ones when the method is applied to samples presenting similar absorption at the wavelength of the light source used in Raman spectroscopy or by performing Raman analysis with multiwavelength excitations.

The Raman spectra were then further analysed in order to understand the observed activity trend in terms of the present defect sites. Indeed, soot combustion is believed to proceed through a Mars-van Krevelen oxygen vacancy mediated mechanism, similarly to CO oxidation. The two suggested pathways of the reaction involve (i) the formation of reactive oxygen O_2_^x−^ on reduced Ce^3+^ or reduced substitutional cations associated with the presence of oxygen vacancies, followed by spillover towards the soot particles, or (ii) the reduction of the catalyst through the direct transfer of lattice oxygen at the soot-ceria interface, which occurs together with oxygen vacancies formation^65^. All the three defect sites identified by Raman spectroscopy can be fruitfully involved in such mechanisms. D1 Frenkel pairs contribute to oxygen mobility and transfer^66^ in the presence of reducing species. D2 defects containing a doping cation foster the weakening of lattice oxygen bonds. Finally, the D3 sites, associated with reduced Ce^3+^ or foreign cations, are representative of the reducibility of the catalyst and can be readily activated to form O_2_^x−^. In particular, the higher D3/F_2g_ ratio for the Cu-containing catalysts suggests a higher reducibility of these samples, which is associated with a lower vacancy formation energy^61^, and is confirmed by the best CO oxidation activity^31^. Moreover, a higher soot conversion at low temperature (<350 °C) was detected in standard combustion tests for these catalysts in comparison to CeO_2_ and Ce95Mn5 (Supplementary Fig. S7a), which could be related to the consumption of the already present D3 sites (their involvement in O_2_^x−^ species formation and consequent oxidation) in the initial heating stage. However, the lack of a correlation between the D/F_2g_ ratio and the soot combustion activity above T_5%_ evidences differences between the CO and soot oxidation reactions. Several factors have been cited in the previous literature to explain this phenomenon. First of all, the lack of a dependence of soot conversion on the surface area of the catalyst was noticed and attributed to the importance of soot-catalyst contact points, rather than of the whole available surface area^58^. This trend seems to be confirmed by the set of the four catalysts, where no clear dependence on the surface area is present, which was instead previously observed for CO oxidation. In agreement with this hypothesis, the well-defined nanocubic morphology of the undoped ceria has been previously shown to provide advantages in terms of soot oxidation activity^31^. Such effect seems to be mostly related to the increased number of catalyst-soot contact points with respect to the small polyhedra agglomerates, since the abundancy of the highly active (100) facets^31^ was not higher for the CeO_2_ and Ce95Mn5 nanocubes compared to the Ce95Cu5 and Ce95Cu2.5Mn2.5 samples.

Moreover, it was recently postulated that the presence of a large amount of oxygen vacancies can lead to reactive oxygen species deactivation through their reduction, favouring the formation of new lattice oxygen instead of highly active peroxides and superoxides^58,67^. Such phenomenon can be particularly detrimental in the case of soot oxidation, where the regeneration of oxygen vacancy sites is limited by the few contact points with the reductant (soot particles). Based on these hypotheses, it can be suggested that in the case of soot oxidation the presence of a higher content of oxygen vacancies in the Cu-doped catalysts does not allow the regeneration of all the consumed reduced defect sites and therefore the formation of new O_2_^x−^ species. The capture of reactive oxygen species by vacancies may compete with soot oxidation, leading to the deactivation of part of the defect sites. Despite their crucial role, no Raman bands attributable to peroxides and superoxides, which are usually detected in the 830–860 cm^−1^ and 1125–1139 cm^−1^ range on ceria based materials^50,68,69^, could be observed on the catalysts, nor at RT neither in the first heating phase. It should be noted that their features have been rarely directly observed at RT^69^, while highly reducing conditions, such as H_2_ atmosphere at elevated temperature followed by cooling in oxygen, are usually required for their stable formation. Instead, they probably only exist as transient species in the here analysed catalytic process, especially above 250 °C, where their Raman signal results strongly attenuated according to TPD (Temperature Programmed Desorption) experiments on nanostructured ceria materials performed by Wu *et al*.^50^ Interestingly, the mechanism of vacancy annihilation by oxygen species proposed by them to explain the desorption of only the 20% of the adsorbed oxygen species recalls the described vacancy-O_2_^x−^ reaction, showing that this is actually a significant pathway in ceria catalysts.

**Figure 8:**
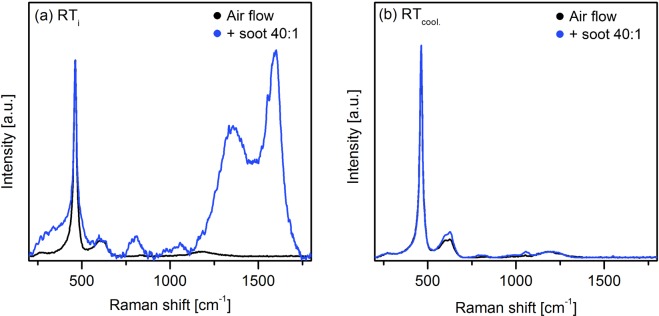
Comparison between the RT Raman spectra recorded on the tablets of Ce95Cu5 during the soot oxidation test and the analysis in air flow, at the beginning (**a**) and at the end (**b**) of the test. All the spectra were normalized to the F_2g_ band.

However, an influence of such mechanism on the defect sites could be expected. Figure 8 shows the comparison between the RT Raman spectra recorded on the Ce95Cu5 catalyst-soot tablets during the *in situ* test and those previously obtained on the pure catalyst in air flow at the beginning (Fig. 8a) and at the end (Fig. 8b) of the tests. The spectra were normalized to the F_2g_ peak of ceria. Before heating, the spectrum is extremely noisy, thus a reliable analysis of the D bands is not possible. Instead, at the end of the test, when the shielding effect of soot is not present anymore, the defects band in the spectrum of the tablet is clearly more intense than that of the spectrum recorded on the pure catalyst. The huge increase of the D1 and especially the D2 band of Ce95Cu5 observed after soot conversion may be indicative of the partial conversion of vacancy sites to oxidized sites, supporting the conclusions drawn above. Table 4 summarizes the D/F_2g_ ratios calculated for the Ce95Cu5 sample using the spectra recorded at RT at the beginning and at the end of the different carried out tests. The D/F_2g_ ratio increases during each test, but the increment is definitely higher when soot oxidation takes place on the catalyst; this reaction seems to enhance the formation of new defects and to promote structural changes. Similar comparisons between the RT_cool._ spectra recorded during the *in situ* soot oxidation tests and during the analyses in air flow for the other three catalysts are reported in Supplementary Fig. S10. A slight increase of the D2 band is observed in the case of Ce95Cu2.5Mn2.5 too, consistently with its low soot oxidation activity, while the curves are almost overlapped for the CeO_2_ and Ce95Mn5 samples, showing that if any change in the defect sites occurs during soot conversion, such variation is reversible. It seems therefore that the higher soot oxidation activity of the last two catalysts is not only related to their nanocubic morphology, but also to their ability to avoid O_2_^x−^ species deactivation, which can be instead indirectly observed in the Raman spectra of the Cu-containing catalysts through the increase of the D2 component at the end of the tests.

**Table 4:** D/F_2g_ ratios at RT for the Ce95Cu5 sample before and after the different types of test.

Type of test	D/F_2g_		
	RT_i_	RT_cool._	Variation
Static	0.310	0.357	+15.1%
Air flow	0.196	0.228	+16.6%
Soot oxidation	0.305	0.449	+47.0%

Thus, pure and Mn-doped ceria, which display a stable defect sites distribution, are characterized by the highest activity in soot oxidation while Ce95Cu5 and partially Ce95Cu2.5Mn2.5 undergo important changes in the defect types during soot combustion. The results indicate that Mn can be profitably added to ceria catalysts, since it improves the physico-chemical properties with beneficial effects on the activity during the soot oxidation.

## Conclusions

In the current work, four different ceria-based catalysts (CeO_2_, Ce95Cu5, Ce95Mn5 and Ce95Cu2.5Mn2.5), prepared through hydrothermal synthesis, were studied by *in situ* Raman spectroscopy to monitor the presence and the evolution of defect sites at high temperatures and during the soot oxidation reaction. Cu and Mn doping fostered the formation of both intrinsic and extrinsic defects in the ceria framework, whose concentration was estimated by the Raman band intensity ratio Dn/F_2g_. In detail, two main Raman components were identified in the defects band: the D1 peak around 600 cm^−1^, assigned to intrinsic Frenkel anion pairs, and the D2 component around 630 cm^−1^, ascribed to extrinsic defect sites. The higher reducibility of the Cu-containing catalysts was supported by the detection of an additional weak band around 560 cm^−1^, due to reduced Ce^3+^ associated with oxygen vacancies.

High temperature cycles in air provided no evidence for new oxygen vacancies formation, while the observed changes were mostly due to the combination of strain and anharmonicity effects and were reversible at RT, except for Ce95Cu5, for which the extrinsic defect band increased its intensity. Tests in different atmospheres suggested the assignment of the D2 band to oxidized Cu-containing sites without oxygen vacancies.

*In situ* Raman analyses on soot-catalyst tablets allowed to directly observe soot conversion in the range between 375 to 450 °C, depending on the studied catalyst, both from the decrease of carbon Raman bands and from the huge rise in intensity of ceria features due to the gradually reduced absorption of light by soot. A method was proposed to calculate the soot conversion from Raman spectra and the resulting trends were compared to those obtained with standard methods: Ce95Mn5 was confirmed as the best catalyst for soot oxidation, while less agreement was found among the conversion curves of the other samples, due to their different light-absorption features.

The almost reversible behaviour of pure and Mn-doped ceria even after soot conversion demonstrated the superior ability of these catalysts to regenerate the most active defect sites. Instead, a huge increase of both D1/F_2g_ and D2/F_2g_ ratios in case of Ce95Cu5 at the end of the test, which was already evident after the complete conversion of soot, was observed; this result is compatible with the deactivation of reactive oxygen species through their reaction with oxygen vacancies. Such an effect is less marked but present also in case of the Ce95Cu2.5Mn2.5 catalyst, suggesting that the formation of oxygen vacancy-free defect sites is promoted during soot oxidation on defective catalysts presenting a huge quantity of oxygen vacancies on their surface. These outcomes provide new insights into the role of the defects in the catalytic combustion of soot, proving that *in-situ* Raman analyses can support the design of more active catalysts.

## Methods

### Catalyst preparation

In the present work, four different catalysts were prepared: the CeO_2_ sample is made of pure ceria, while the other samples, named Ce95Cu5, Ce95Mn5 and Ce95Cu2.5Mn2.5, are made of ceria doped with 5% Cu, 5% Mn, and 2.5% Cu together with 2.5% Mn, respectively. All the catalysts were obtained via hydrothermal synthesis, with a procedure reported elsewhere^31^. Appropriate amounts of Ce(NO_3_)_3_·6H_2_O (Sigma-Aldrich) and Cu(NO_3_)_2_·5H_2_O (Sigma-Aldrich) and/or Mn(NO_3_)_2_·4H_2_O (Sigma-Aldrich) were dissolved in a beaker containing 10 ml of deionized water, stirring the solution for some minutes. Separately, another solution was prepared by adding 48 g of NaOH in 70 ml of deionized water. The nitrate-containing solution was then slowly added drop by drop to the second solution. The mixture thus obtained was stirred for 1 h at RT, then it was poured into a 150 ml Teflon liner, which was placed in an autoclave. Subsequently, an aging treatment was carried out by heating the autoclave in an oven at 180 °C for 24 h. The precipitate thus obtained was rinsed several times, using alternately deionized water and ethanol to remove impurities. The precipitate was then placed in an oven in which it was dried at 60 °C overnight. Finally, the dry powder obtained was gently crushed in a mortar and calcined in an oven at 550 °C for 4 h.

### Catalyst characterization

All the four synthesized catalysts were studied using different characterization techniques, to identify the main physico-chemical and morphological characteristics of each sample^31^.

Powder X-ray diffraction was performed in a Philips X’Pert PW3040 diffractometer, using a Cu K*α* radiation (wavelength *λ* = 1.5418 · 10^−10^ m, 2*θ* range = 20°–70°; step = 0.05° 2*θ*; time per step = 0.2 s). The diffraction peaks were indexed according to the Powder Data File database (PDF 2000, International Centre of Diffraction Data, Pennsylvania). The crystal average size *D*_c_ (nm) was estimated using Scherrer’s equation, *D*_c_ = 0.9 λ/(*β* cos *θ*), where 0.9 is the shape factor for spheres, *λ* is the Cu K*α* wavelength (nm), *β* is the full-width at half maximum FWHM (rad) and *θ* is the Bragg angle (rad). The FWHM data were previously corrected comparing them with those of a lanthanum hexaboride standard.

The BET specific surface area (S_BET_) and the total pore volume (V_p_) of the catalysts were measured through nitrogen physisorption at −196 °C in a Micromeritics ASAP 2020, via the Brunauer-Emmett-Teller method. The mean pore diameter (D_p_) was also estimated from the adsorption isotherm using the Barrett-Joyner-Halenda algorithm. Prior to the analysis, the samples were outgassed at 200 °C for 2 h in order to eliminate any gases adsorbed on the surface.

The samples were observed with a field emission scanning electron microscope (FESEM) Zeiss Merlin with a Gemini-II column, in order to study their morphology. A thin Cr layer was deposited on the samples to improve the conductivity, before acquiring the micrographs.

The catalysts were investigated via X-ray photoelectron spectroscopy using a PHI VersaProbe apparatus, with a band-pass energy of 187.85 eV, a take-off angle of 45° and an X-ray spot diameter of 100 μm.

Diffuse reflectance UV-Visible-NIR spectra of the catalysts were collected in the 200–1500 nm with an Agilent CARY 5000 spectrophotometer.

### Raman analyses

Raman spectroscopy was employed to study surface defectiveness. All Raman measurements were performed using a Renishaw InVia Reflex micro-Raman spectrometer, equipped with a 100 mW power solid-state laser emitting monochromatic light at a wavelength of 514.5 nm. The spectra, obtained with 15 accumulations lasting 15 seconds each under a 5x objective, were carefully analyzed with the Renishaw software WiRE 3.4, in order to perform the fitting and deconvolution of the peaks and to calculate for each of them position, intensity, width and area. Except for the pure ceria catalyst, three Lorentzian curves were used to fit the D band, whose centers were initially set at the typical Raman shift reported by the previous literature for the different defect sites. The final contribution (null for the D3 peak for some catalysts) and the parameters of each curve were determined by the software optimization algorithm (using the Root Mean Square method). The *D*/*F*_2g_ value, representative of the defects density, was calculated as the ratio between the integrated areas of the deconvoluted defects-related Raman bands (D) and the main vibrational component related to the ceria lattice (*F*_2g_). RT measurements and the relative deconvolution were repeated on three spectra for each catalyst, showing reproducible results.

#### Static analyses at high temperature

These analyses were carried out using a Linkam TS1500 cell, inserted in the Raman spectrometer and connected to a temperature controller. Some powder was placed in a tungsten crucible, inserted in the Linkam cell. After having recorded a spectrum at RT (RT_i_), the cell was heated up to 100 °C with a rate of 10 °C/min, and it was then left at this temperature for ten minutes before recording a new Raman spectrum. This procedure was similarly repeated for the other considered temperatures, up to 700 °C. Afterwards, the cell was cooled to 350 °C, and after waiting ten minutes a spectrum was collected (350 °C_cool._). Finally, the sample was cooled to RT, and ten minutes later a spectrum was recorded (RT_cool._).

#### Analyses in air flow at high temperature

These analyses were carried out heating the cell with a constant rate of 3 °C/min, continuously, up to 700 °C. Furthermore, a 45 ml/min air flow was sent into the cell during the heating phase, while a 45 ml/min pure nitrogen flow was flushed during the cooling phase. In order to prevent the gas flow from displacing the powder, the tests were performed on catalyst tablets, obtained by compressing some powder into a press. A small portion of a tablet was inserted into the cell crucible, perpendicular to the laser beam. After recording the spectrum at RT (RT_i_), the cell was heated and spectra at various temperatures were collected. Once the 700 °C spectrum had been recorded, the temperature was kept constant and pure nitrogen was fluxed for 15 min; then a new spectrum was acquired at 700 °C, that would allow to detect any changes induced by the variation of the atmosphere. Finally, during the cooling phase two spectra were measured at 350 °C (350 °C_cool._) and after reaching RT (RT_cool._).

#### *In situ* analyses of soot conversion

These measurements were carried out on tablets of catalyst and soot, prepared by mixing adequate quantities of catalyst and Printex U particulate (Degussa) in a ball mill and then compressing the resulting powder in a press. In this way, “tight” contact conditions were obtained, with a high degree of contact between the two solids. A catalyst-soot weight ratio of 40:1 was chosen, so that the Raman signal of the soot did not completely cover the peaks associated to the presence of defects in the catalyst. A 45 ml/min air flux was sent into the cell during both the heating and cooling phases. After all the soot in the tablet had been converted, the cell was cooled and two spectra were measured at 350 °C (350 °C_cool._) and at RT (RT_cool._).

## Supplementary information

Supplementary information

## Data Availability

The datasets generated during the current study are available from the corresponding author on reasonable request.
