# Supplementary material for: In situ Raman analyses of the soot oxidation reaction over nanostructured ceria-based catalysts
Source: Sci Rep. 2019 Mar 7;9:3875. doi: 10.1038/s41598-019-39105-5 (PMC6405916; doi:10.1038/s41598-019-39105-5)
Supplement: Supplementary file 1 — Supplementary information [file 41598_2019_39105_MOESM1_ESM.pdf]

Supplementary information for:

In situ Raman analyses of the soot oxidation reaction over nanostructured ceria-based catalysts

Enrico Sartoretti[1+], Chiara Novara[1+], Fabrizio Giorgis[1], Marco Piumetti[1], Samir Bensaid[1*], Nunzio Russo[1], Debora Fino[1]

[1]Department of Applied Science and Technology, Politecnico di Torino, Corso Duca degli Abruzzi, 24, 10129 Turin, Italy. Correspondence and requests for materials should be addressed to S.B. (email: samir.bensaid@polito.it)

+these authors contributed equally to this work

Catalyst characterization

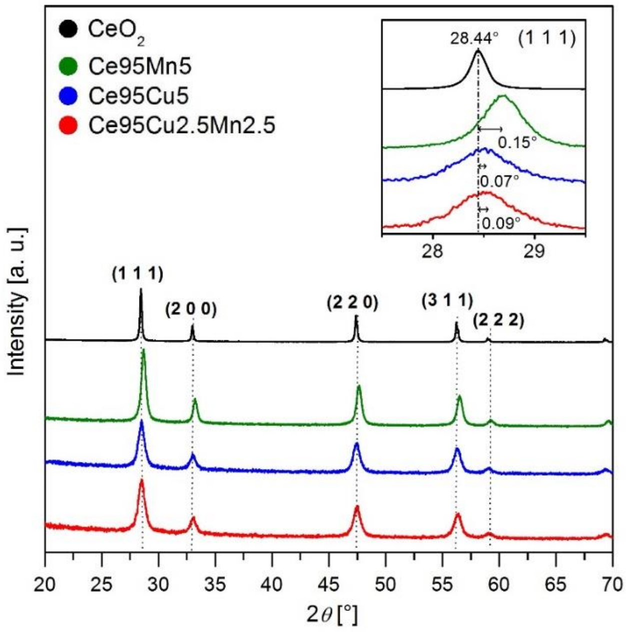

Supplementary Figure S1. XRD diffractograms of the prepared samples, with a magnification of the (1 1 1) peak. All the powder XRD diffractograms exhibit the typical peaks of the crystalline fluorite structure of ceria, characterized by peaks related to the (1 1 1), (2 0 0), (2 2 0), (3 1 1) and (2 2 2) planes. The magnification of the (1 1 1) peak in the 2θ range from 28° to 29° is shown in the inset, in which a shift to higher 2θ values is visible for the doped samples. These displacements are probably due to ceria lattice shrinkage, since the ionic radii of copper and manganese are smaller than those of cerium. The largest shift of the (1 1 1) peak is about 0.15 ° and occurs for the Ce95Mn5 catalyst, since manganese ions have the smallest radii among the ions of the elements taken into account; minor displacements of about 0.07 ° and 0.09 ° are visible in Ce95Cu5 and Ce95Cu2.5Mn2.5 diffractograms respectively. (XRD, except for Ce95Mn5, reported in [31]).

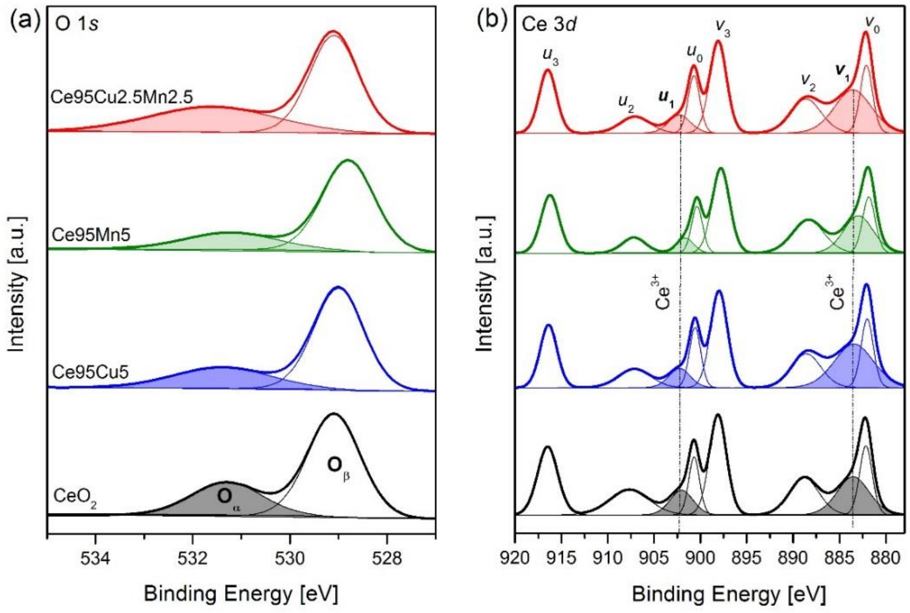

Supplementary Figure S2. XPS spectra of the four samples in the O 1*s* and Ce 3*d* core levels. The traces obtained by curve fit and deconvolution of the bands are also reported (XPS, except for Ce95Mn5, reported in [31]).

Raman analyses

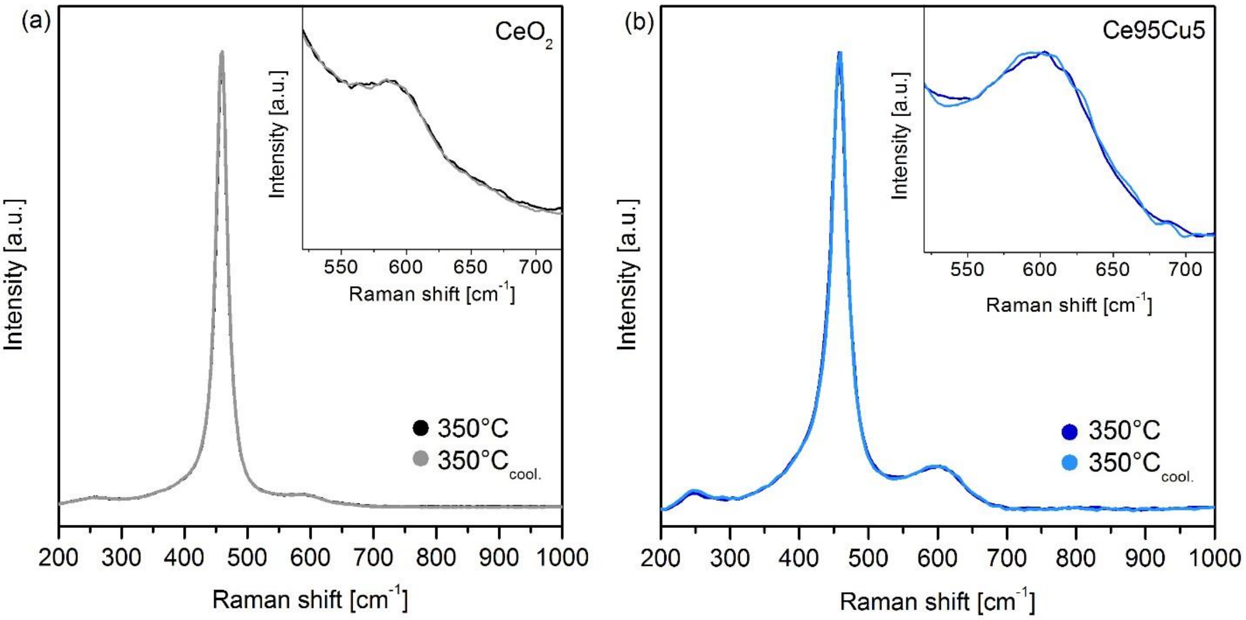

Supplementary Figure S3. Comparison between the Raman spectra collected at 350 °C during the heating and cooling phase of the static tests on CeO_2 (a) and Ce95Cu5 (b); in the insets, magnifications of the defects band region are shown. All the spectra were normalized to the F_{2g} band.

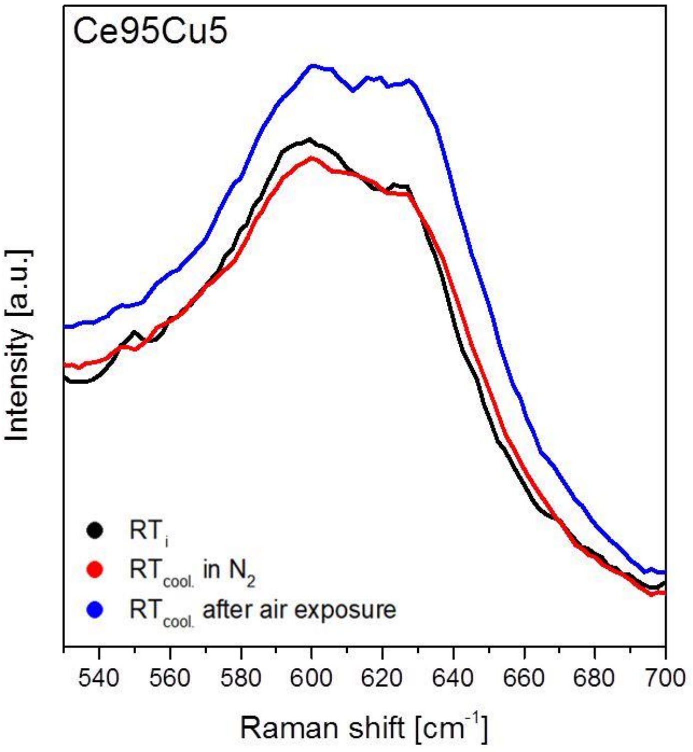

Supplementary Figure S4. Magnification of the defects region of Raman spectra collected at RT on a tablet of Ce95Cu5. After pure nitrogen was fluxed into the cell, the first spectrum (RT_i) was acquired. The temperature was increased with a rate of 3 °C/min until 700 °C, then the sample was cooled down to RT in N_2 atmosphere and a spectrum was collected ($RT_{cool.}$ in N_2). Finally, air was fluxed into the cell and after 10 min a new spectrum was detected ($RT_{cool.}$ after air exposure). All the spectra were normalized to the F_{2g} band.

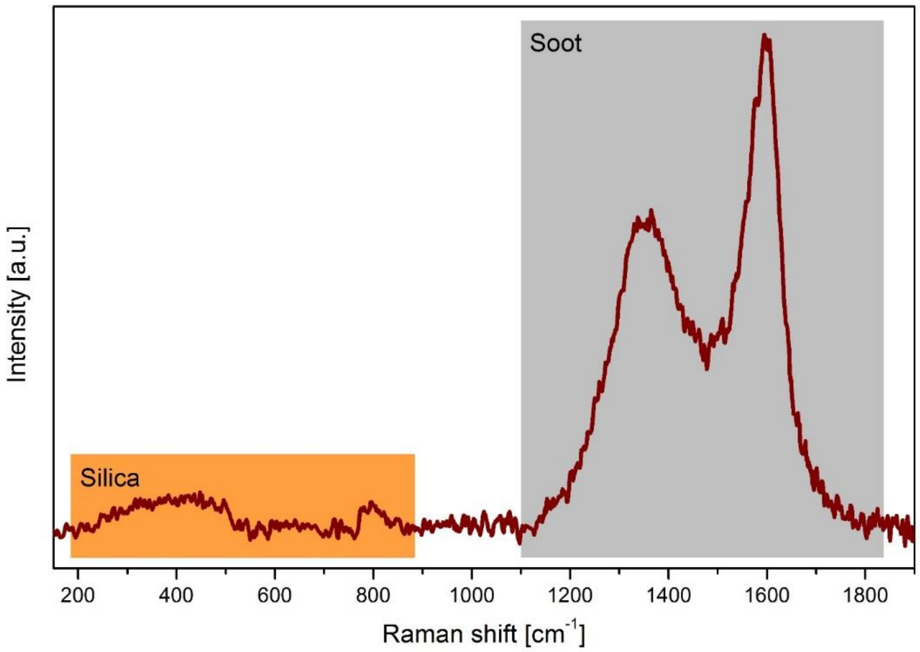

Supplementary Figure S5. Raman spectra collected at RT on a tablet of pure Printex U, in which silica bands are visible.

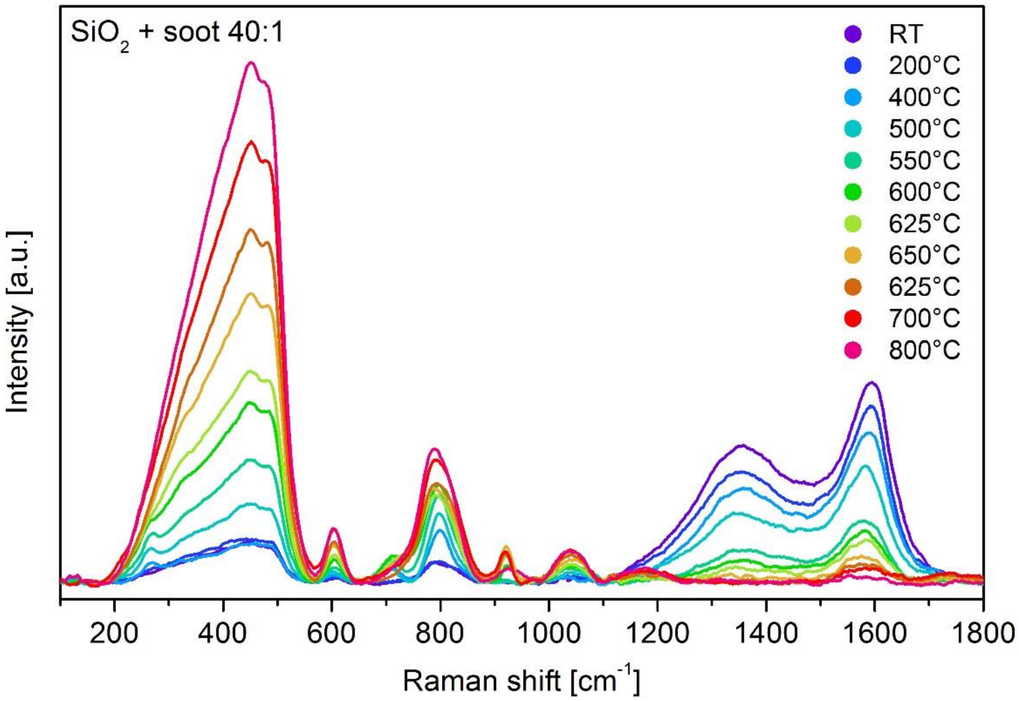

Supplementary Figure S6. Raman spectra collected at different temperatures during the in situ soot oxidation test on a 40:1 silica-soot tablet. Silica features gain intensity when soot is oxidized, similarly to the ceria bands of the soot-catalyst tablets; therefore, the integrated area of silica band from 200 to 550 cm^{-1} was exploited to calculate the Raman based conversion curve for the soot-silica tablet reported in Fig. 7b.

Catalytic performance

Oxidation tests were carried out to investigate the performance of the four catalysts during the conversion of soot. A mixture of 45 mg of powder catalyst, 5 mg of Printex U particulate (Degussa) and 150 mg of silica was prepared in a ball mill (Giuliani Tecnologie), using a speed of 290 rpm for 15 min, to obtain "tight contact" condition, i.e. a high degree of contact among the different solids. A fixed bed reactor was prepared depositing the mixed powders on a quartz wool support inserted in a quartz U-tube reactor (ID = 4 mm, OD = 6 mm); a K-type thermocouple was placed into the U-tube, with the sensor tip as close as possible to the reactor bed, and then the tube was housed in a PID-controlled electric furnace. The bed was initially pre-treated for 30 min at 100 °C with a flow of 100 ml/min of air. Subsequently, the test was started by introducing in the reactor a gas containing 50% of air and 50% of nitrogen by volume, with a flow of 100 ml/min. The temperature was gradually increased from 100 °C up to 700 °C, with a rate of 5 °C/min. The soot conversion as a function of temperature, reported in Supplementary Fig. S7a, was calculated by monitoring the quantities of CO and CO_2 (Supplementary Fig. S7b and S7c) produced in the reactor with a non-dispersive infrared analyser (ABB Uras 14). Temperatures corresponding to 10%, 50% and 90% conversion (denoted as $T_{10\%}$, $T_{50\%}$ and $T_{90\%}$, respectively) were taken as indices of activity, and are reported in Supplementary Table S1.

The inset in Supplementary Fig. S7a shows the magnification of the curves between 260 and 340 °C. In this range, soot conversion appears higher for Ce95Cu2.5Mn2.5 and Ce95Cu5 and this is probably related to their higher specific surface area and the initial availability of reduced D3 sites, as detailed in the successive discussion. Above 400 °C, the Ce95Mn5 sample is the most performing catalyst in the series, since the trend of $T_{50\%}$ follows this order: Ce95Mn5 < CeO_2 < Ce95Cu5 < Ce95Cu2.5Mn2.5. At high temperature the effect of morphology seems to overcome the effect of dopants and a structure-sensitive behaviour can be observed. The nanocubic structure of Ce95Mn5 and CeO_2, characterized by reactive low-index planes, apparently plays an important role in soot oxidation, as already supposed in previous investigations[11,35].

All these catalysts are able not only to lower the reaction temperature but also to enhance the selectivity of the reaction towards CO_2, as can be concluded observing Supplementary Fig. S7b and S7c, which show the CO and CO_2 concentrations produced during the soot oxidation tests. CeO_2 is the most unselective catalyst as it produces the highest CO concentration, while Ce95Cu5 is the most selective towards CO_2.

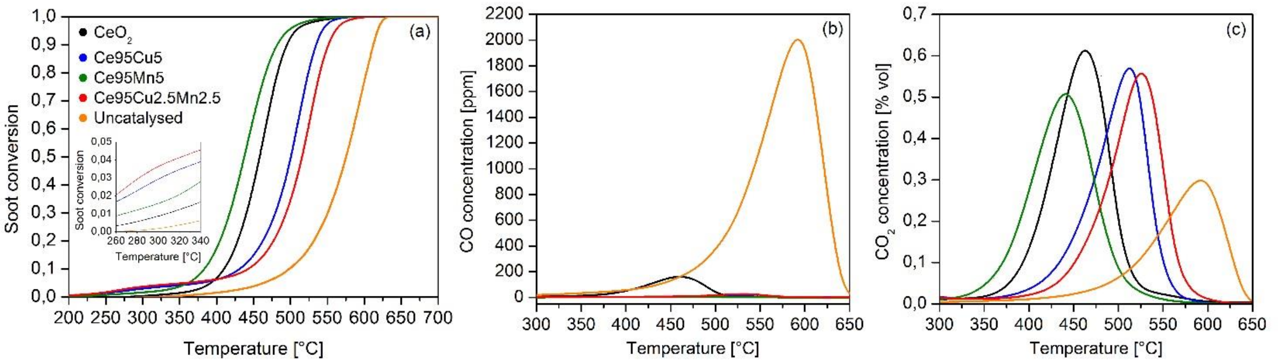

Supplementary Figure S7. Conversion of soot as a function of temperature during the oxidation tests (a), with a magnification of the curves between 260 and 340 °C in the inset; concentration of CO (b) and CO_2 (c) produced during the soot oxidation tests (detailed catalysts' activity assessment, except for Ce95Mn5, reported in [31]).

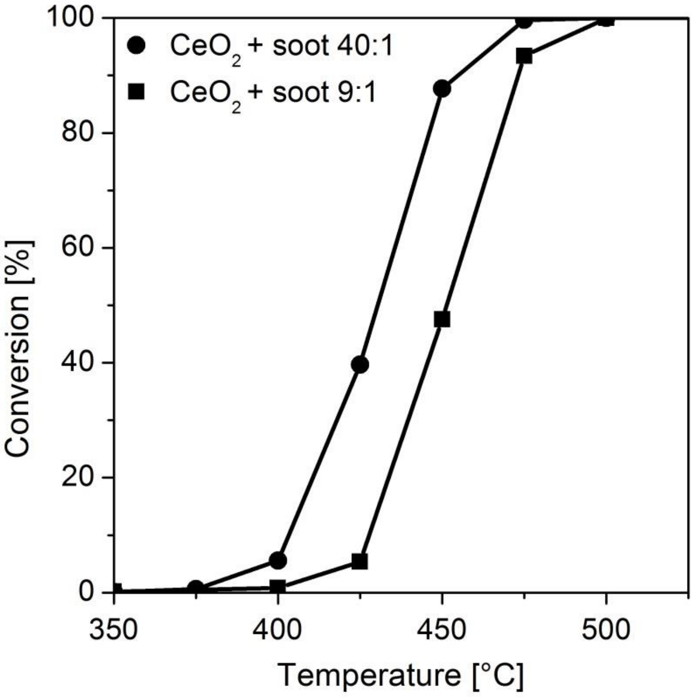

Supplementary Figure S8. Comparison between the conversion curves obtained from the Raman spectra collected during the in situ analysis of soot oxidation on tablets with a 40:1 and 9:1 catalyst-soot ratio.

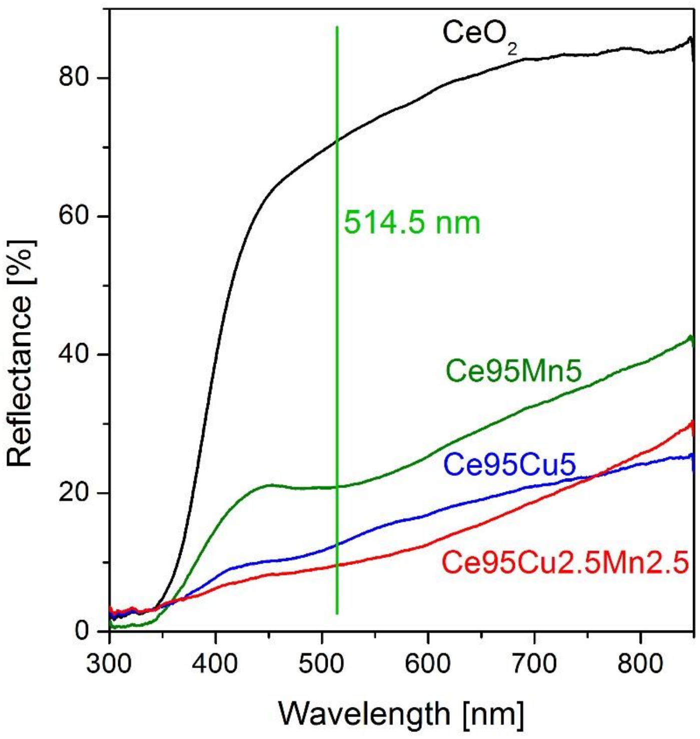

Supplementary Figure S9. Diffuse reflectance UV-Vis-NIR spectra collected on the CeO_2, Ce95Cu5, Ce95Mn and Ce95Cu2.5Mn2.5 catalysts. The green line marks the 514.5 nm excitation laser wavelength employed for the Raman measurements.

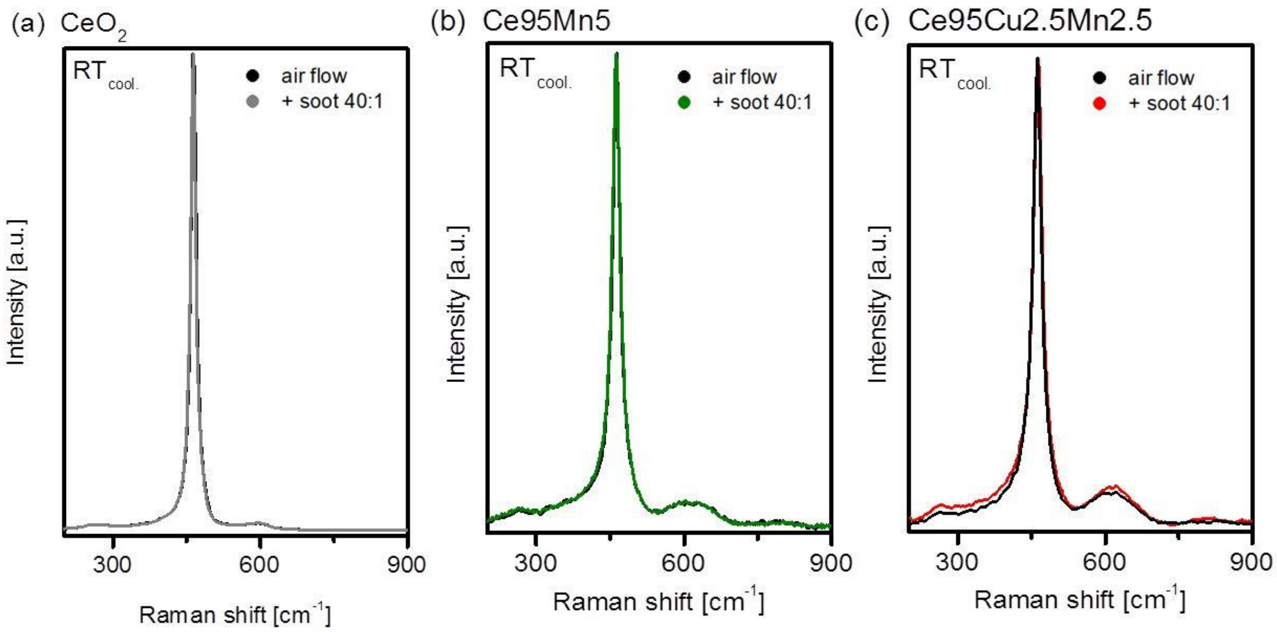

Supplementary Figure S10. Raman spectra collected at RT at the end of the analyses in air flow and of the in situ tests with soot on the CeO_2 (a), Ce95Mn5 (b) and Ce95Cu2.5Mn2.5 (c) samples. All the spectra were normalized to the F_{2g} band.

Catalyst	$T_{10\%}$ [°C]	$T_{50\%}$ [°C]	$T_{90\%}$ [°C]
CeO_2	409	455	494
Ce95Cu5	430	503	533
Ce95Mn5	382	436	481
Ce95Cu2.5Mn2.5	441	516	552

Supplementary Table S1. Catalytic performance parameters obtained from the soot oxidation tests (detailed catalysts' activity assessment, except for Ce95Mn5, reported in [31]).
